# Neighboring Gene Regulation by Antisense Long Non-Coding RNAs

**DOI:** 10.3390/ijms16023251

**Published:** 2015-02-03

**Authors:** Victoria E. Villegas, Peter G. Zaphiropoulos

**Affiliations:** 1 Department of Biosciences and Nutrition, Karolinska Institutet, Huddinge 14183, Sweden; E-Mail: peter.zaphiropoulos@ki.se; 2 Faculty of Natural Sciences and Mathematics & Doctoral Program in Biomedical Sciences, Universidad del Rosario, Bogotá 11001000, Colombia

**Keywords:** regulatory RNA, antisense transcription, long non-coding RNAs, gene regulation

## Abstract

Antisense transcription, considered until recently as transcriptional noise, is a very common phenomenon in human and eukaryotic transcriptomes, operating in two ways based on whether the antisense RNA acts in *cis* or in *trans*. This process can generate long non-coding RNAs (lncRNAs), one of the most diverse classes of cellular transcripts, which have demonstrated multifunctional roles in fundamental biological processes, including embryonic pluripotency, differentiation and development. Antisense lncRNAs have been shown to control nearly every level of gene regulation—pretranscriptional, transcriptional and posttranscriptional—through DNA–RNA, RNA–RNA or protein–RNA interactions. This review is centered on functional studies of antisense lncRNA-mediated regulation of neighboring gene expression. Specifically, it addresses how these transcripts interact with other biological molecules, nucleic acids and proteins, to regulate gene expression through chromatin remodeling at the pretranscriptional level and modulation of transcriptional and post-transcriptional processes by altering the sense mRNA structure or the cellular compartmental distribution, either in the nucleus or the cytoplasm.

## 1. Introduction

Emerging evidence suggests that genomes transcribe a much larger repertoire of non-coding compared to protein-coding RNAs, a phenomenon that is more elaborate in complex organisms [1]. Additionally, the number and types of known functional non-coding RNAs, short or long in size, has been significantly expanded, as these may be involved in *cis* or *trans* regulation of genes located in their vicinity or at distant loci [2]. Within these non-coding RNAs, a class transcribed from the antisense strand of well-defined transcriptional units, the antisense non-coding RNAs, were initially considered as transcriptional noise, due to low evolutionary conservation and low levels of expression. However, there is now considerable evidence that antisense transcripts act in almost all stages of gene expression, from transcription and translation to RNA degradation [3,4]. Moreover, antisense long non-coding RNAs (lncRNAs) can be involved in the regulation of the expression of either their neighboring genes in *cis* or more distant genes in *trans* through various mechanisms.

The focus of this article is centered on functional studies of antisense lncRNA-mediated regulation of neighboring gene expression in mammals, especially humans, with particular emphasis on the interaction mechanisms and types of regulation, *i.e.*, pretranscriptional, transcriptional or posttranscriptional.

## 2. Antisense Transcription

Antisense transcription is defined as transcription from the opposite strand of a protein-coding gene or a sense strand-derived RNA [5]. It is a very common phenomenon in human and eukaryotic transcriptomes [6,7,8]. Several studies have demonstrated that more than 63% of transcripts have antisense partners, many of which are not encoding proteins, with the majority being expressed at lower levels than the sense RNA [5,9,10,11,12]. Antisense transcription operates in two ways based on whether the antisense RNA acts in *cis* or in *trans*. In *cis*, the antisense RNA interacts with a gene transcribed from the same DNA region, whereas in *trans*, the interaction is with genes located at distant loci or even at other chromosomes [13,14].

**Figure 1:**
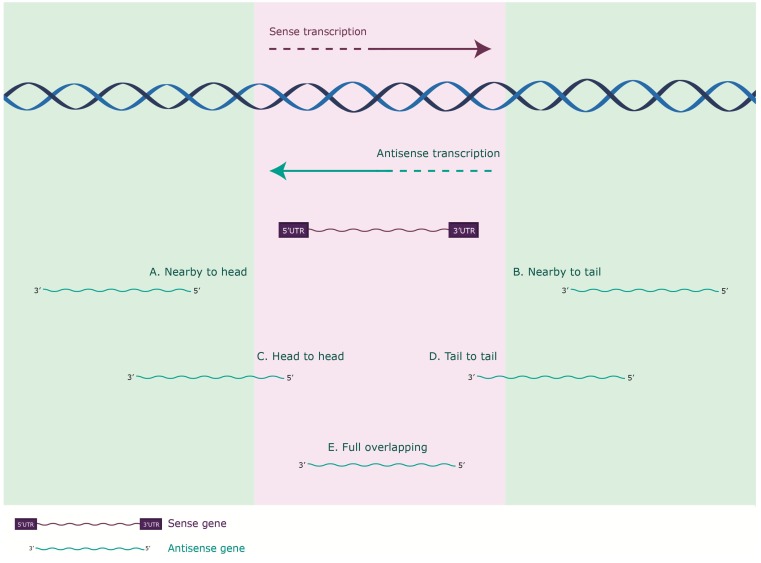
Categorization of *cis* antisense transcription according to the proximity between the sense coding (purple) and antisense non coding (green) genes in the genome. (**A**) Nearby to head, 5' end of an antisense gene is near the 5' end of a sense; (**B**) Nearby to tail, 3' end of a sense gene is near the 3' end of an antisense (these two forms are also known as intergenic); (**C**) Hear to head or divergent, 5' end of both genes align together; (**D**) Tail to tail or convergent, 3' ends of both genes align together; and (**E**) Full overlapping where the antisense gene completely overlaps the sense gene.

*Cis* antisense transcription can be further categorized according to the proximity between the sense and antisense partners in the genome (Figure 1) and classified as: nearby to head, when the 5' end of the sense gene is near the 5' end of the antisense; nearby to tail, when the 3' end of the sense gene is near the 3' end of the antisense gene, these two forms also known as intergenic [7,15]; head-to-head or divergent, when the 5' ends of both the sense and antisense genes align together; tail-to-tail or convergent, when the 3' ends of both the sense and antisense genes align together and full overlapping when the sense gene completely overlaps with the antisense one [16,17,18].

## 3. Long Non-Coding RNA

About 2% of the human genome is transcribed into mature protein-coding RNAs, while the large majority, between 70%–90%, is transcribed into non-protein coding RNAs (ncRNAs). ncRNAs include several types of RNA, exemplified by transfer RNAs (tRNAs), ribosomal RNAs (rRNAs) and small nuclear RNAs (snRNAs), with very well defined function in many cellular processes [11,19,20,21]. Besides these classical ncRNAs, there are various ncRNAs of unknown function, many of which are considered as lncRNAs based on their size greater being than 200 nucleotides [19,22]. These, apart from the lack of protein-coding potential, have relatively low levels of evolutionary conservation and originate from genes that are usually shorter than protein-coding genes, with fewer exons [12,22]. On the other hand, they exhibit certain similarities with protein-coding transcripts; for example, they are typically transcribed by RNA polymerase II and can be capped, polyadenylated and spliced. Their cellular localization can be either in the nucleus or the cytoplasm, with the former being consistent with the major function of lncRNAs as epigenetic modulators [20,23,24].

Although most lncRNAs share similarities with mRNAs regarding transcript processing, the 5' cap structure and 3' poly(A) tail, recently identified lncRNAs highlighted alternative processing mechanisms, for example capping by small nucleolar RNAs (snoRNAs) at both ends or forming circular RNA structures. Classes of lncRNAs that lack a poly(A) tail include: eRNAs (enhancer RNAs); sno-lncRNAs (snoRNA-related lncRNAs); circRNAs (circular RNAs); and ciRNAs (circular intronic RNAs) [25]. Yin* et al.* identified lncRNAs derived from excised introns, flanked at both ends by intronic small nucleolar RNA sequences (sno-lncRNAs), which are specifically expressed in pluripotent cells. The most abundant sno-lncRNAs, from chromosomal region 15q11–q13, interacted with alternative splicing regulator FOX2 (feminizing locus on X) conferring changes in FOX2-dependent splicing, with a limited *cis* effect on the 15q11–q13 locus, [26]. Moreover, it has been clearly established that the expression of sno-lncRNAs is species specific and that their processing is closely linked to alternative splicing of their parental gene [27]. Another type of lncRNAs with non-canonical 5' and 3' ends are the circular RNAs that are formed either by back-splicing of exons, circRNAs [28,29], or derived from excised introns, ciRNAs [30]. circRNAs are largely localized to the cytoplasm, and recent studies have revealed that they may function as efficient “sponges”, sequestering microRNAs and consequently regulating gene expression [28,29]. On the other hand, ciRNAs are retained in the nucleus, have little enrichment for microRNA target sites and may be involved in modulating the expression of the host gene, features that distinguish them from circRNAs [30].

The recent research emphasis on lncRNAs has demonstrated multifunctional roles in fundamental biological processes, including embryonic pluripotency, differentiation and development [31,32]. Dysregulation of lncRNAs has also been associated with a broad range of physiological defects. Silver–Russell, Turner, Prader–Willi and HELLP (hemolysis, elevated liver enzymes, and low platelets in the mother) syndromes are some representative examples [33,34,35,36]. Additionally, cardiac diseases, myopathies, neurodegenerative disorders and cancers have also been linked with lncRNAs [31,37,38,39,40].

The H19 lncRNA has been characterized as an oncogene in hepatocellular and bladder carcinoma, as well as in colon and breast cancer. More recently, increased H19 levels have been found to be associated with high grade gliomas, and H19 depletion inhibits the invasion of glioma cells [41,42]. The maternally expressed gene 3 (*MEG3*) lncRNA has also been correlated with gliomas, but in contrast to H19, the MEG3 levels are markedly decreased in gliomas, while its overexpression inhibits cell proliferation and promotes apoptosis* in vitro* [43,44].

According to a study using full-length cDNA datasets from human and mouse, lncRNAs may originate from various genomic positions, but predominantly from the vicinity of protein-coding genes. This suggests that transcription of certain lncRNAs may depend on the same promoter regions as the nearby protein-coding genes [45].

Bioinformatics analysis is indicative of structural conformations of lncRNAs that may be biologically relevant. For example, the function of the MEG3 and SRA1 lncRNAs, as a tumor suppressor and a hormone receptor coactivator, respectively, is apparently maintained through interactions between distinct secondary structural elements [39,46].

## 4. Mechanisms of Regulation Mediated by Antisense lncRNA

Compared with coding transcripts, most antisense lncRNAs are expressed at 10-fold lower levels on average, and their expression in different tissues and cell types has generally been found to be more cell type specific [22,32]. A biological role of antisense lncRNAs, despite this low expression, could still be rationalized due to the fact that there are two copies of DNA for any given gene in a cell; consequently, just two antisense lncRNA molecules are sufficient to interact with the two gene copies and elicit regulatory effects [47]. Recent studies monitoring the half-life of lncRNAs have demonstrated higher stability for antisense and spliced forms, which opens the possibility to postulate that versatile molecular functions may depend on this biochemical feature [22,48].

Antisense lncRNAs are functionally very diverse [7,39]. They can act as positive and negative modulators of protein-coding genes [7,49,50], regulators of gene expression, involved in diverse functions, such as X inactivation [51,52], imprinting, epigenetic regulation [24,53,54] and can affect any step within the biogenesis or mobilization of the target RNA, including transcription, mRNA splicing, nuclear and cytoplasmic trafficking and translation [20]. LncRNAs can impact genes in the same chromosomal locus or in other chromosomes; however, this review focuses on antisense lncRNAs that modify the expression of neighboring genes.

**Figure 2:**
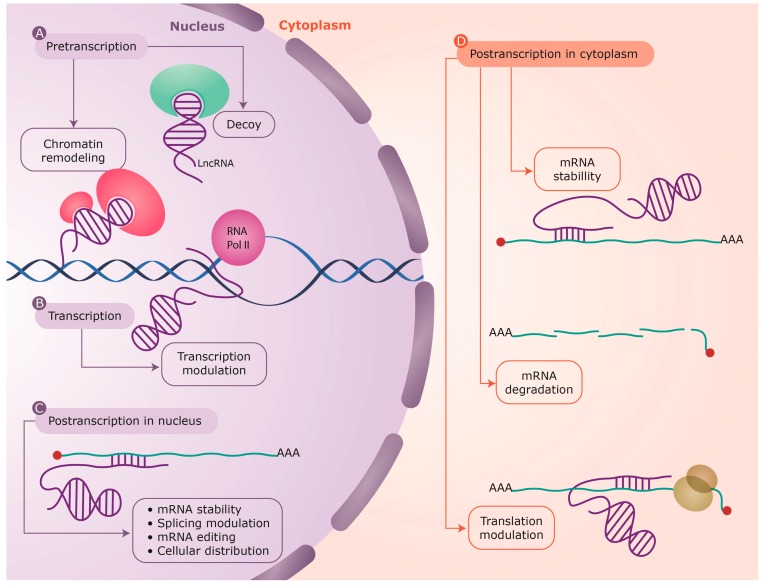
Antisense IncRNAs have been found to act at nearly every level of gene regulation: (**A**) Pretranscriptional, as protein guides or acting as decoys holding proteins away from chromatin; (**B**) Transcriptional, as modulators of transcription; (**C**,**D**) Posttranscriptional, altering sense mRNA structure or cellular compartmental distribution either in the nucleus or the cytoplasm. LncRNAs are depicted in purple, with the interacting protein factors in green and light red. The mRNAs are shown as green lines and the base pair interactions highlighted by short purple lines. Also shown is the transcribing RNA polymerase II (RNA Pol II) on genomic DNA (blue helix) and the translating ribosome (yellow) on the mRNA.

Antisense lncRNAs have been found to act at nearly every level of gene regulation [22,31,55]: pretranscriptionally (Figure 2A), as guides of proteins into specific parts of the genome, as decoys keeping proteins away from chromatin and through epigenetic changes by histone modifications or DNA cytosine methylation [46]; transcriptionally (Figure 2B), conferring modulatory effects in the transcriptional process [56]; and posttranscriptionally (Figure 2C,D), through RNA-RNA interactions that alter mRNA structure or cellular compartmentalization, either in the nucleus or the cytoplasm [31,48]. The versatile regulatory functions of lncRNAs fall into different categories, depending on the interacting partner (Table 1), as follows: lncRNA-DNA, lncRNA–RNA and lncRNA–protein interactions [12]. These are further discussed below.

### 4.1. DNA-RNA Interaction

Thousands of lncRNAs have emerged as key molecular players in epigenetic processes through their association with chromatin modifiers [46]. Specifically, antisense RNAs in the nucleus can act as regulators of their counterpart sense mRNA by modulating chromatin structure in *cis* and by bridging epigenetic effectors and regulatory complexes at specific genomic loci [24].

Sequence complementarity can establish complex configurations, such as RNA–DNA duplexes and triplexes. These direct RNA–DNA (Table 1A) interactions could efficiently and selectively target RNA signals to genomic loci [12]. These signals may influence the DNMT3 family of DNA methyl transferases, which induces *de novo* DNA methylation, the Polycomb repressive complex PRC2, which elicits histone H3 lysine 27 trimethylation (H3K27me3), or G9a/GLP methyltransferases targeting histone H3 lysine 9 (H3K9), which correlate with transcriptional repression [32,46,57].

There is currently limited evidence for widespread direct interactions between lncRNAs and DNA through the formation of RNA:DNA hybrids or triplex structures that are based on sequence complementarity [46]. However, there are some very well-characterized examples, as follows:

RASSF1A (RAS-association domain family member 1A, RAS comes from rat sarcoma) is one of eight different transcripts generated by alternative splicing/alternative promoter usage. It encodes a protein similar to the RAS effector proteins, with multiple modulatory functions at apoptotic and cell cycle checkpoint pathways. Additionally, its inactivation is implicated in the development of many human cancers [58]. ANRASSF1 (antisense intronic non-coding RASSF1) is a capped and polyadenylated unspliced long non-coding RNA, with nuclear localization, which is transcribed in the antisense direction relative to the protein-coding mRNAs of the *RASSF1* gene locus, maps upstream of the RASSF1C isoform and overlaps most of the of RASSF1 isoforms. This transcript is expressed in several cell lines and tissues, and its interaction with genomic DNA, forming an RNA/DNA hybrid*,* leads to downregulation of the sense gene at the pretranscriptional level. ANRASSF1 recruits PRC2 to the *RASSF1A* promoter, then PRC2 induces the accumulation of the repressive mark H3K27me3, which confers a specific reduction in the RASSF1A transcriptional activity. Current research has not detected any effect of ANRASSF1 on the expression of the RASSF1C isoform, which is under the control of a different promoter, or the levels of H3K27me3 and PRC2 recruitment at the *RASSF1C* promoter and at the promoters of neighboring genes in the *RASSF1* locus, demonstrating the selectivity of its regulatory mechanism [59].

**Table 1:** LncRNA mechanisms of action based on molecular interaction with nucleic acids (**A**,**B**) and protein (**C**), adapted by permission from [12].

Molecular Interaction	Description/Some Examples	References
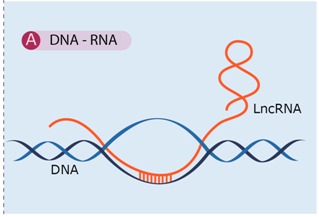	LncRNAs can establish complex configurations as RNA–DNA duplexes and triplexes, which can associate with regulatory proteins to affect neighboring regions. This regulation mechanism operates at pretranscriptional and transcriptional level.	[46,57,59,60,61,62,63]
	Examples: ANRASSF1, pRNA (promoter associated RNA), ANRIL (antisense non-coding RNA in the INK4 locus).	
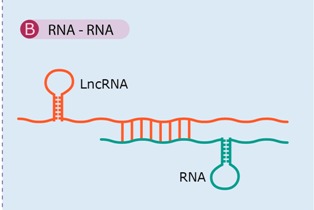	Sense RNA and antisense lncRNA transcripts can hybridize and form RNA duplexes and this interaction results in dfferent posttranscriptional outcomes, all of which modulate sense mRNA expression.	[47,64,65,66,67]
	Examples: Antisense Uchl1, BACE1-AS, PTENpg1.	
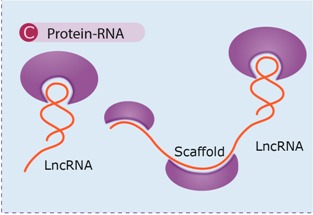	lncRNAs can function as molecular decoys retaining proteins and preventing their function or acting as platforms for the assembly of protein complexes (scaffold) through distinct protein-binding domains. This regulation mechanism operates at all levels in gene expression.	[68,69,70,72]
	Examples: Xist, APOA1-AS, GLl1AS.	

Chromatin remodeling complex NoRC silences a subset of *rRNA* genes by recruiting chromatin-modifying enzymes to the *rDNA* promoter. It requires for its function a heterogeneous population of 150–300-nucleotide RNA, termed pRNA (promoter-associated RNA), in an activity that coordinates several macromolecular complexes to modify histones and methylate DNA [60]. pRNA transcripts, originating from the intergenic spacer that separates *rRNA* genes, are non-coding RNAs in sense or antisense orientation that are complementary to the *rDNA* promoter, with numerous potential functions, including promoter-targeted RNA-induced transcriptional gene regulation via *de novo* CpG methylation of *rRNA* genes [60,61].

Specifically, these non-coding RNAs interact with complementary sequences within the rDNA promoter forming a RNA–DNA–DNA triplex [73] that is specifically recognized by the DNA methyltransferase DNMT3b, thereby inducing DNA methylation and transcriptional silencing [74,75]. Indeed, the pRNA triplex structure with DNA displaces the transcription factor, TTF-I, from its binding site [73].

*ANRIL* (antisense non-coding RNA in the *INK4* locus) spans a region of 126.3 kb and is transcribed in the antisense orientation of the *INK4b-ARF-INK4a* gene cluster, which plays a central role in cell cycle inhibition, senescence and stress-induced apoptosis [76]. This antisense RNA is transcribed by RNA polymerase II and is spliced into several isoforms, most of which are polyadenylated, with some reported to be tissue-specific with low expression, as is the case of other functional non-coding RNAs [77,78,79]. The *INK4b-ARF-INK4a* locus is located on the human chromosome 9p21 and encodes three tumor-suppressor proteins, p15INK4b, p14ARF and p16INK4a [76,80]. The first intron of *ANRIL* overlaps with the two exons of *p15INK4b* and maintains the silencing state of this gene. The 5' end of the *ANRIL* first exon is located −300 bp upstream of the transcription start site (TSS) of the *p14ARF* gene, suggesting that these two genes may share a bidirectional promoter [62,63]. *ANRIL* was shown to be involved in epigenetic regulation of the *INK4b-ARF-INK4A* locus by recruiting Polycomb repressive complex-1 (PRC1) and -2 (PRC2) to form heterochromatin surrounding the *INK4b-ARF-INK4a* locus, leading to repression of gene expression [77]. The nascent ANRIL lncRNA associates with Suz12 to recruit the PRC2 complex and initiate H3K27me3, then recruits PRC1, providing another docking site for H3K27me3 binding, which results in the maintenance of epigenetic repression [54,62,76]. *ANRIL* expression is regulated by ATM-E2F1 signaling, and alteration in its structure and/or expression mediates the susceptibility to a variety of chronic diseases and cancer predisposition [81,82].

### 4.2. RNA–RNA Interaction

Sense RNA and antisense lncRNA transcripts can hybridize and form RNA duplexes (Table 1B) by virtue of their ability to base pair. Consequently, lncRNAs can act as highly specific sensors of mRNA, with this interaction resulting in different posttranscriptional outcomes, all of which modulate sense mRNA expression; In fact, through such interactions, lncRNAs can interfere with the splicing, RNA editing, subcellular distribution, transport or nuclear retention of the corresponding sense RNA transcripts. Moreover, nuclear and cytoplasmic sense-antisense hybrids can alter mRNA stability and modulate translation [38,47].

Carrieri* et al.* found that an antisense lncRNA transcribed from the opposite strand of the mouse *Uchl1* (ubiquitin carboxy-terminal hydrolase L1) gene in a head-to-head orientation can activate polysomes for increased translation of Uchl1 under certain stress conditions [64]. The posttranscriptional control (Figure 2C,D) of the coding gene is mediated through a SINEB2 element, which overlaps with the 5' end of the *Uchl1* gene, including the AUG codon, through RNA–RNA interactions between the sense and antisense transcripts (Table 1B). Consistently, overexpression of the antisense RNA results in increased expression of the Uchl1 protein without any increase in Uchl1 mRNA levels [64].

Beta-secretase 1 or β-site amyloid precursor protein cleaving enzyme 1 (BACE1) is a protease that participates in the sequential cleavage of amyloid precursor protein, a crucial enzyme in Alzheimer’s disease (AD) pathophysiology. Faghihi* et al.* identified a conserved lncRNA, BACE1-AS, transcribed from the opposite strand of the *BACE1* locus (11q 23.3), with 104 nucleotides of full complementarity to exon 6 of the human BACE1 mRNA, which regulates protein expression of the sense gene in the brain of an AD mouse model [83]. The BACE1-antisense transcript promotes the stability of the BACE1 mRNA through the formation of RNA duplexes, leading to the production and deposition of amyloid-β peptide (Aβ) and the deleterious feed-forward cycles of disease progression [65]. The characterization of this antisense RNA has allowed the initiation of new studies, which postulate that silencing the expression of the endogenous lncRNA BACE1-AS can diminish Aβ formation and neuronal damage [65,66].

*PTEN* is a tumor-suppressor gene in chromosome 10q23 that is mutated in a large number of human cancers [84] and is posttranscriptionally regulated in *trans* through an intricate mechanism involving a pseudogene, known as *PTENpg1*, located in 9p23, and a non-coding RNA antisense to this pseudogene, which is termed PTENpg1as [50].

The PTENpg1 sense RNA lacks a poly(A) tail, and its stability and export to the cytoplasm is facilitated by interactions with PTENpg1as. There are three PTENpg1as variants: alpha, beta and unspliced. Alpha and beta are spliced, polyadenylated isoforms found predominately in the cytoplasm, whereas the unspliced isoform is exclusively found in the nucleus [50]. PTENpg1as alpha physically localizes to the *PTEN* promoter and functions as an epigenetic modulator by the recruitment of DNMT3a and enhancer of zeste. On the other hand, PTENpg1as beta forms an RNA–RNA duplex with the PTENpg1 transcript, altering the subcellular distribution and increasing PTENpg1 RNA stability. This enhances the miRNA sponge-like properties of PTENpg1 and, ultimately, its capacity to posttranscriptionally regulate PTEN [50,67].

### 4.3. RNA–Proteins and Scaffolds

lncRNAs are mainly associated with proteins due to the possession of distinct protein-binding domains. Such complexes may have an impact on transcription, acting as key regulators of gene expression. lncRNAs can function as molecular decoys, with sponge-like properties, taking away proteins from a specific location or recruiting proteins to activate or inhibit processes involved at any level of gene regulation. Miscellaneous RNA–protein interactions can support the assembly of protein complexes (scaffold), which link the factors together in order to generate brand new functions that may impact the process of transcription [43,68], either positively or negatively (Table 1C).

In mammals, X inactivation avoids the dosage differences of X-linked genes between males and females, as one of the two copies of the X chromosome present in female cells is inactivated by Xist [51]. This gene is located on the X chromosome and is transcribed as a long non-protein-coding RNA with a transcript size of 17 kb in humans. Xist binds in *cis* and exerts transcriptional repression on the entire X chromosome through DNA and histone methylation, global histone hypoacetylation and the Polycomb repressive complex, PRC2 [52]. This lncRNA interacts with its chromatin targets via YY1, a bivalent protein, capable of binding both RNA and DNA through different sequence motifs; YY1-Xist-PRC2 (the scaffold model in Table 1C) forms a nucleation center, which spreads in *cis* along the X chromosome [69,70].

Recent studies have found that *apolipoprotein A1* (*APOA1*), a gene with a specific role in lipid metabolism, expresses a long non-coding antisense transcript, APOA1-AS, which modulates the expression of not only APOA1, but also multiple neighboring genes,* in vitro* and* in vivo*, acting as a modular scaffold and negative transcriptional regulator. This antisense transcript has two exons and spans two neighboring genes, *APOA1* and *SIK3* (*Salt-inducible kinase 3*). APOA1-AS acts via recruitment of H3K27me3 marks along the promoter regions of *APOA1*, *APOA4* and *APOC3*, facilitating the interaction of the histone-modifying enzymes, LSD1 and SUZ12, and the PRC2 complex [71].

An additional antisense lncRNA with a scaffold-type regulatory impact is GLI1AS, which generates repressive chromatin marks at its locus, acting as an epigenetic modifier that negatively regulates the expression of neighboring genes [72]. Among these, *GLI1* (*glioma-associated oncogene 1*) encodes a transcription factor that is a marker of Hedgehog signaling activation, and its increased expression is associated with a wide variety of human cancers [85]. GLI1AS is located head-to-head with the *GLI1* gene and tail-to-tail with the *INHBE* gene. Its gene repressive effects via H3K27me3 appear to be more pronounced on the *GLI1* than the *INHBE* gene [72].

## 5. Conclusions

The accumulated evidence suggests that the complexity of organisms is associated with an intricate network of gene regulatory processes. Consequent to a better understanding of these mechanisms and their molecular contexts is the growing research focus in this direction.

Long non-coding RNAs with an antisense orientation toward known protein-coding genes are demonstrated to significantly contribute to the repertoire of regulatory mechanisms that are used by mammalian cells to modulate gene expression. These may depend on base pair complementarity or on their ability to bind to proteins, thereby serving as platforms and/or adaptors for diverse DNA–RNA, RNA–RNA or RNA–protein interactions.

Admittedly, our understanding of the mechanistic details of the biological function of antisense long non-coding RNAs is still in the early stages, yet it is clear that they can act at nearly all levels of gene regulation, including transcription, mRNA processing and translation.

## References

[1] Kapranov P., Willingham A.T., Gingeras T.R. (2007). Genome-wide transcription and the implications for genomic organization. Nat. Rev. Genet..

[2] Guil S., Esteller M. (2012). *Cis*-acting noncoding RNAs: Friends and foes. Nat. Struct. Mol. Biol..

[3] Pelechano V., Steinmetz L.M. (2013). Gene regulation by antisense transcription. Nat. Rev. Genet..

[4] Yang L., Froberg J.E., Lee J.T. (2014). Long noncoding RNAs: Fresh perspectives into the RNA world. Trends Biochem. Sci..

[5] Katayama S., Tomaru Y., Kasukawa T., Waki K., Nakanishi M., Nakamura M., Nishida H., Yap C.C., Suzuki M., Kawai J. (2005). Antisense transcription in the mammalian transcriptome. Science.

[6] Györffy A., Surowiak P., Tulassay Z., Györffy B. (2007). Highly expressed genes are associated with inverse antisense transcription in mouse. J. Genet..

[7] Numata K., Kiyosawa H. (2012). Genome-wide impact of endogenous antisense transcripts in eukaryotes. Front. Biosci..

[8] Yelin R., Dahary D., Sorek R., Levanon E.Y., Goldstein O., Shoshan A., Diber A., Biton S., Tamir Y., Khosravi R. (2003). Widespread occurrence of antisense transcription in the human genome. Nat. Biotechnol..

[9] Carninci P., Kasukawa T., Katayama S., Gough J., Frith M.C., Maeda N., Oyama R., Ravasi T., Lenhard B., Wells C. (2005). The transcriptional landscape of the mammalian genome. Science.

[10] Li K., Ramchandran R. (2010). Natural antisense transcript: A concomitant engagement with protein-coding transcript. Oncotarget.

[11] Nishizawa M., Okumura T., Ikeya Y., Kimura T. (2012). Regulation of inducible gene expression by natural antisense transcripts. Front. Biosci..

[12] Guttman M., Rinn J.L. (2012). Modular regulatory principles of large non-coding RNAs. Nature.

[13] Brantl S. (2007). Regulatory mechanisms employed by *cis*-encoded antisense RNAs. Curr. Opin. Microbiol..

[14] Chen J., Sun M., Kent W.J., Huang X., Xie H., Wang W., Zhou G., Shi R.Z., Rowley J.D. (2004). Over 20% of human transcripts might form sense-antisense pairs. Nucleic Acids Res..

[15] Osato N., Suzuki Y., Ikeo K., Gojobori T. (2007). Transcriptional interferences in *cis* natural antisense transcripts of humans and mice. Genetics.

[16] Werner A. (2013). Biological functions of natural antisense transcripts. BMC Biol..

[17] Wood E.J., Chin-Inmanu K., Jia H., Lipovich L. (2013). Sense-antisense gene pairs: Sequence, transcription, and structure are not conserved between human and mouse. Front. Genet..

[18] Soldà G., Suyama M., Pelucchi P., Boi S., Guffanti A., Rizzi E., Bork P., Tenchini M.L., Ciccarelli F.D. (2008). Non-random retention of protein-coding overlapping genes in Metazoa. BMC Genomics.

[19] Atkinson S.R., Marguerat S., Bähler J. (2012). Exploring long non-coding RNAs through sequencing. Semin. Cell Dev. Biol..

[20] Chen L.-L., Carmichael G.G. (2010). Decoding the function of nuclear long non-coding RNAs. Curr. Opin. Cell Biol..

[21] Dinger M.E., Amaral P.P., Mercer T.R., Mattick J.S. (2009). Pervasive transcription of the eukaryotic genome: Functional indices and conceptual implications. Brief. Funct. Genomic Proteomic.

[22] Clark B.S., Blackshaw S. (2014). Long non-coding RNA-dependent transcriptional regulation in neuronal development and disease. Front. Genet..

[23] Bergmann J.H., Spector D.L. (2014). Long non-coding RNAs: Modulators of nuclear structure and function. Curr. Opin. Cell Biol..

[24] Magistri M., Faghihi M.A., St Laurent G., Wahlestedt C. (2012). Regulation of chromatin structure by long noncoding RNAs: Focus on natural antisense transcripts. Trends Genet..

[25] Zhang Y., Yang L., Chen L.-L. (2014). Life without A tail: New formats of long noncoding RNAs. Int. J. Biochem. Cell Biol..

[26] Yin Q.-F., Yang L., Zhang Y., Xiang J.-F., Wu Y.-W., Carmichael G.G., Chen L.-L. (2012). Long noncoding RNAs with snoRNA ends. Mol. Cell.

[27] Zhang X.-O., Yin Q.-F., Wang H.-B., Zhang Y., Chen T., Zheng P., Lu X., Chen L.-L., Yang L. (2014). Species-specific alternative splicing leads to unique expression of sno-lncRNAs. BMC Genomics.

[28] Hansen T.B., Jensen T.I., Clausen B.H., Bramsen J.B., Finsen B., Damgaard C.K., Kjems J. (2013). Natural RNA circles function as efficient microRNA sponges. Nature.

[29] Memczak S., Jens M., Elefsinioti A., Torti F., Krueger J., Rybak A., Maier L., Mackowiak S.D., Gregersen L.H., Munschauer M. (2013). Circular RNAs are a large class of animal RNAs with regulatory potency. Nature.

[30] Zhang Y., Zhang X.-O., Chen T., Xiang J.-F., Yin Q.-F., Xing Y.-H., Zhu S., Yang L., Chen L.-L. (2013). Circular intronic long noncoding RNAs. Mol. Cell.

[31] Batista P.J., Chang H.Y. (2013). Long noncoding RNAs: Cellular address codes in development and disease. Cell.

[32] Fatica A., Bozzoni I. (2014). Long non-coding RNAs: New players in cell differentiation and development. Nat. Rev. Genet..

[33] Cocchi G., Marsico C., Cosentino A., Spadoni C., Rocca A., de Crescenzo A., Riccio A. (2013). Silver-Russell syndrome due to paternal H19/IGF2 hypomethylation in a twin girl born after* in vitro* fertilization. Am. J. Med. Genet. A.

[34] Van Dijk M., Thulluru H.K., Mulders J., Michel O.J., Poutsma A., Windhorst S., Kleiverda G., Sie D., Lachmeijer A.M.A., Oudejans C.B. (2012). HELLP babies link a novel lincRNA to the trophoblast cell cycle. J. Clin. Investig..

[35] Stelzer Y., Sagi I., Yanuka O., Eiges R., Benvenisty N. (2014). The noncoding RNA IPW regulates the imprinted DLK1-DIO3 locus in an induced pluripotent stem cell model of Prader–Willi syndrome. Nat. Genet..

[36] Rajpathak S.N., Vellarikkal S.K., Patowary A., Scaria V., Sivasubbu S., Deobagkar D.D. (2014). Human 45,X fibroblast transcriptome reveals distinct differentially expressed genes including long noncoding RNAs potentially associated with the pathophysiology of Turner syndrome. PLoS One.

[37] Scheuermann J.C., Boyer L.A. (2013). Getting to the heart of the matter: Long non-coding RNAs in cardiac development and disease. EMBO J..

[38] Vučićević D., Schrewe H., Orom U.A. (2014). Molecular mechanisms of long ncRNAs in neurological disorders. Front. Genet..

[39] Li C.H., Chen Y. (2013). Targeting long non-coding RNAs in cancers: Progress and prospects. Int. J. Biochem. Cell Biol..

[40] Kornfeld J.-W., Brüning J.C. (2014). Regulation of metabolism by long, non-coding RNAs. Front. Genet..

[41] Shi Y., Wang Y., Luan W., Wang P., Tao T., Zhang J., Qian J., Liu N., You Y. (2014). Long non-coding RNA H19 promotes glioma cell invasion by deriving miR-675. PLoS One.

[42] Luo M., Li Z., Wang W., Zeng Y., Liu Z., Qiu J. (2013). Long non-coding RNA H19 increases bladder cancer metastasis by associating with EZH2 and inhibiting E-cadherin expression. Cancer Lett..

[43] Park J.Y., Lee J.E., Park J.B., Yoo H., Lee S.-H., Kim J.H. (2014). Roles of long non-coding RNAs on tumorigenesis and glioma development. Brain Tumor Res. Treat..

[44] Wang P., Ren Z., Sun P. (2012). Overexpression of the long non-coding RNA MEG3 impairs* in vitro* glioma cell proliferation. J. Cell Biochem..

[45] Khachane A.N., Harrison P.M. (2010). Mining mammalian transcript data for functional long non-coding RNAs. PLoS One.

[46] Mercer T.R., Mattick J.S. (2013). Structure and function of long noncoding RNAs in epigenetic regulation. Nat. Struct. Mol. Biol..

[47] Faghihi M.A., Wahlestedt C. (2009). Regulatory roles of natural antisense transcripts. Nat. Rev. Mol. Cell Biol..

[48] Wu P., Zuo X., Deng H., Liu X., Liu L., Ji A. (2013). Roles of long noncoding RNAs in brain development, functional diversification and neurodegenerative diseases. Brain Res. Bull..

[49] Su W.-Y., Li J.-T., Cui Y., Hong J., Du W., Wang Y.-C., Lin Y.-W., Xiong H., Wang J.-L., Kong X. (2012). Bidirectional regulation between WDR83 and its natural antisense transcript DHPS in gastric cancer. Cell Res..

[50] Johnsson P., Ackley A., Vidarsdottir L., Lui W.-O., Corcoran M., Grandér D., Morris K.V. (2013). A pseudogene long-noncoding-RNA network regulates *PTEN* transcription and translation in human cells. Nat. Struct. Mol. Biol..

[51] Brown C.J., Hendrich B.D., Rupert J.L., Lafrenière R.G., Xing Y., Lawrence J., Willard H.F. (1992). The human *XIST* gene: Analysis of a 17 kb inactive X-specific RNA that contains conserved repeats and is highly localized within the nucleus. Cell.

[52] Lee J.T. (2009). Lessons from X-chromosome inactivation: Long ncRNA as guides and tethers to the epigenome. Genes Dev..

[53] Gupta R.A., Shah N., Wang K.C., Kim J., Horlings H.M., Wong D.J., Tsai M.-C., Hung T., Argani P., Rinn J.L. (2010). Long non-coding RNA HOTAIR reprograms chromatin state to promote cancer metastasis. Nature.

[54] Tollervey J.R., Lunyak V.V. (2012). Epigenetics: Judge, jury and executioner of stem cell fate. Epigenetics.

[55] Faust T., Frankel A., D’Orso I. (2012). Transcription control by long non-coding RNAs. Transcription.

[56] Vance K.W., Ponting C.P. (2014). Transcriptional regulatory functions of nuclear long noncoding RNAs. Trends Genet..

[57] Hung T., Chang H.Y. (2010). Long noncoding RNA in genome regulation: Prospects and mechanisms. RNA Biol..

[58] Donninger H., Vos M.D., Clark G.J. (2007). The RASSF1A tumor suppressor. J. Cell Sci..

[59] Beckedorff F.C., Ayupe A.C., Crocci-Souza R., Amaral M.S., Nakaya H.I., Soltys D.T., Menck C.F.M., Reis E.M., Verjovski-Almeida S. (2013). The intronic long noncoding RNA ANRASSF1 recruits PRC2 to the RASSF1A promoter, reducing the expression of RASSF1A and increasing cell proliferation. PLoS Genet..

[60] Mayer C., Schmitz K.-M., Li J., Grummt I., Santoro R. (2006). Intergenic transcripts regulate the epigenetic state of rRNA genes. Mol. Cell.

[61] Bierhoff H., Schmitz K., Maass F., Ye J., Grummt I. (2010). Noncoding transcripts in sense and antisense orientation regulate the epigenetic state of ribosomal RNA genes. Cold Spring Harb. Symp. Quant. Biol..

[62] Aguilo F., Zhou M.-M., Walsh M.J. (2011). Long noncoding RNA, polycomb, and the ghosts haunting INK4b-ARF-INK4a expression. Cancer Res..

[63] Pasmant E., Sabbagh A., Vidaud M., Bièche I. (2011). *ANRIL*, a long, noncoding RNA, is an unexpected major hotspot in GWAS. FASEB J..

[64] Carrieri C., Cimatti L., Biagioli M., Beugnet A., Zucchelli S., Fedele S., Pesce E., Ferrer I., Collavin L., Santoro C. (2012). Long non-coding antisense RNA controls *Uchl1* translation through an embedded SINEB2 repeat. Nature.

[65] Liu T., Huang Y., Chen J., Chi H., Yu Z., Wang J., Chen C. (2014). Attenuated ability of BACE1 to cleave the amyloid precursor protein via silencing long noncoding RNA BACE1-AS expression. Mol. Med. Rep..

[66] Modarresi F., Faghihi M.A., Patel N.S., Sahagan B.G., Wahlestedt C., Lopez-Toledano M.A. (2011). Knockdown of BACE1-AS nonprotein-coding transcript modulates β-amyloid-related hippocampal neurogenesis. Int. J. Alzheimers Dis..

[67] Poliseno L., Salmena L., Zhang J., Carver B., Haveman W.J., Pandolfi P.P. (2010). A coding-independent function of gene and pseudogene mRNAs regulates tumour biology. Nature.

[68] Ray D., Kazan H., Cook K.B., Weirauch M.T., Najafabadi H.S., Li X., Gueroussov S., Albu M., Zheng H., Yang A. (2013). A compendium of RNA-binding motifs for decoding gene regulation. Nature.

[69] Jeon Y., Lee J.T. (2011). YY1 tethers Xist RNA to the inactive X nucleation center. Cell.

[70] Chapman A.G., Cotton A.M., Kelsey A.D., Brown C.J. (2014). Differentially methylated CpG island within human XIST mediates alternative P2 transcription and YY1 binding. BMC Genet..

[71] Halley P., Kadakkuzha B.M., Faghihi M.A., Magistri M., Zeier Z., Khorkova O., Coito C., Hsiao J., Lawrence M., Wahlestedt C. (2014). Regulation of the apolipoprotein gene cluster by a long noncoding RNA. Cell Rep..

[72] Villegas V.E., Rahman M.F.-U., Fernandez-Barrena M.G., Diao Y., Liapi E., Sonkoly E., Ståhle M., Pivarcsi A., Annaratone L., Sapino A. (2014). Identification of novel non-coding RNA-based negative feedback regulating the expression of the oncogenic transcription factor GLI1. Mol. Oncol..

[73] Schmitz K.-M., Mayer C., Postepska A., Grummt I. (2010). Interaction of noncoding RNA with the rDNA promoter mediates recruitment of DNMT3b and silencing of rRNA genes. Genes Dev..

[74] Yan B.-X., Ma J.-X. (2012). Promoter-associated RNAs and promoter-targeted RNAs. Cell Mol. Life Sci..

[75] Wehner S., Dörrich A.K., Ciba P., Wilde A., Marz M. (2014). pRNA: NoRC-associated RNA of rRNA operons. RNA Biol..

[76] Kotake Y., Nakagawa T., Kitagawa K., Suzuki S., Liu N., Kitagawa M., Xiong Y. (2011). Long non-coding RNA *ANRIL* is required for the PRC2 recruitment to and silencing of p15(INK4B) tumor suppressor gene. Oncogene.

[77] Yap K.L., Li S., Muñoz-Cabello A.M., Raguz S., Zeng L., Mujtaba S., Gil J., Walsh M. J., Zhou M.-M. (2010). Molecular interplay of the noncoding RNA *ANRIL* and methylated histone H3 lysine 27 by polycomb CBX7 in transcriptional silencing of *INK4a*. Mol. Cell.

[78] Folkersen L., Kyriakou T., Goel A., Peden J., Mälarstig A., Paulsson-Berne G., Hamsten A., Hugh W., Franco-Cereceda A., Gabrielsen A. (2009). Relationship between CAD risk genotype in the chromosome 9p21 locus and gene expression. Identification of eight new *ANRIL* splice variants. PLoS One.

[79] Congrains A., Kamide K., Ohishi M., Rakugi H. (2013). *ANRIL*: Molecular mechanisms and implications in human health. Int. J. Mol. Sci..

[80] Gil J., Peters G. (2006). Regulation of the INK4b-ARF-INK4a tumour suppressor locus: All for one or one for all. Nat. Rev. Mol. Cell Biol..

[81] Chen D., Zhang Z., Mao C., Zhou Y., Yu L., Yin Y., Wu S., Mou X., Zhu Y. (2014). * ANRIL* inhibits p15(INK4b) through the TGFβ1 signaling pathway in human esophageal squamous cell carcinoma. Cell Immunol..

[82] Wan G., Mathur R., Hu X., Liu Y., Zhang X., Peng G., Lu X. (2013). Long non-coding RNA *ANRIL* (CDKN2B-AS) is induced by the ATM-E2F1 signaling pathway. Cell Signal..

[83] Faghihi M.A., Modarresi F., Khalil A.M., Wood D.E., Sahagan B.G., Morgan T.E., Finch C.E., St G., Iii L., Kenny P. J. (2008). Expression of a noncoding RNA is elevated in Alzheimer’ s disease and drives rapid feed-forward regulation of β-secretase. Nat. Med..

[84] Alimonti A., Carracedo A., Clohessy J.G., Trotman L.C., Nardella C., Egia A., Salmena L., Sampieri K., Haveman W.J., Brogi E. (2010). Subtle variations in *Pten* dose determine cancer susceptibility. Nat. Genet..

[85] Hui C.-C., Angers S. (2011). Gli proteins in development and disease. Annu. Rev. Cell Dev. Biol..

